# Epilepsy of Seventeen Years Standing—Trephined

**Published:** 1874-07

**Authors:** A. W. Calhoun

**Affiliations:** Atlanta, Ga.


ATLANTA

MEDICAL AND SURGICAL JOURNAL.

VOL. XII.] JULY—1874. [NO. 4.

### Original Communications.

### EPILEPSY OF SEVENTEEN YEARS STANDING—TREPHINED.

BY A. W. CALHOUN, M.D., ATLANTA, GA.

*Read before the Atlanta Academy of Medicine June* 8, 1874.

On the 5th of March, 1857, more than seventeen years ago, Mr. J. E. M., now of Bartow county, Georgia, at that time a little more than twenty-three years of age, was thrown from an engine by the explosion of the boiler, while employed as an engineer upon the Western & Atlantic Railroad. Whether by the force of the fall—as he was cast some distance—or by a fragment of the locomotive, or whether by striking his head against the sharp edge of a cross-tie which was lying near—whether by any of these the injury was produced is not positively known. Upon examination it was found that a fracture of the skull had taken place over the region of the junction of the frontal and right parietal bones, extending down towards the right temporal. The skin above, and to the outer side of, the right superciliary ridge was torn loose from the bone, somewhat above and a little to one side of the seat of the fracture, as was subsequently demonstrated by the operation. It was thought unadvisable by the attending physicians to interfere further than to cleanse the parts and bring the flesh wound together by means of sutures, adhesive strips, etc., and allow nature to effect a cure, if possible.

For three or four weeks he lay in a semi-conscious state, suffering intense pain during the brief periods of consciousness that now and then supervened. Opiates were given freely, and it is not certainly known whether the injury alone, or the opiates in part, were the cause or causes of this prolonged half comatose condition. He now began slowly to recover, though many months passed before anything like his former health had been regained.

Three months after the injury some symptoms of a convulsion made their appearance, which passed off, to be followed a few months later by an actual epileptic spasm. From this time on epilepsy became a habit with him, being attacked annually with one or two convulsions, each surpassing the previous one in violence, and each leaving the mind in a less settled state.

During the last few years they have become more frequent and more violent, until, for the year preceding the operation, every month brought regularly with it a more exhausting fit than had hitherto attacked the patient. Often, in the intervals between these well developed monthly attacks, epileptic symptoms would appear, similar to those spoken of as appearing soon after the injury had been received. Even these intermediate symptoms were growing more frequent and severe.

After each convulsion the patient suffered "most dreadful headaches," lasting usually twenty-four hours, the pain being referred particularly to the seat of the injury, and especially severe at one point in the depressed portion of bone.

Although feeble, he has been able to continue business in the capacity of engineer upon various railroads, until recently, when, as observed both by himself and friends, his health was so undermined, and his memory and other mental faculties had become so defective, it was no longer thought safe for himself or others to continue in business.

Up to the time of the injury the patient's health had been excellent. He had been told, however, that as a child, while suffering from the presence of worms, he had had a few convulsions. He has himself no recollection of this.

Every conceivable mode of treatment has been practiced, and every kind of doctor (to use the patient's expression), has been consulted for relief, but to no purpose. When I first saw the patient, early in April last, in company with Drs. H.V. M. Miller, J. M. Johnson and W. F. Westmoreland, his face presented that silly, inexpressive look so common in confirmed epileptics; an expression that might well be denominated "epileptic," and which, if I am not mistaken, is rather indicative of epilepsy and kindred affections. His conversation bore out fully the impression produced by his countenance. There was sense in what he said, yet his manner of expression would be at once remarked as being silly; hesitating in relating trivial circumstances, as though he was afraid of not getting properly through with the story. There was great difficulty in carrying himself back in thought to events that had transpired subsequent to the injury. Pressure over the depressed bone was quite painful, and particularly sensitive was one certain spot.

On the 15th April, with the assistance of Drs. Miller and J. M. Johnson, Scott, Kendrick and Griggs, chloroform was given, and a ⊢—— shaped incision was made over the seat of the injury, and the flaps dissected back, leaving the bone bare. The depression was found to be about 1½ inch in length, running from above downwards and backwards, taking in a portion each of the frontal and parietal bones, and extending slightly into the temporal. The bone had the appearance of having been struck with a blunt-edged instrument.

A conical screw trephine was applied directly over the point most sensitive, as mentioned above, in the frontal bone, a little in front of the anterior-inferior angle of the right parietal bone, and a button removed. Upon the removal of this button a good deal of hemorrhage followed, owing to the vessels of this part of the disploe being much enlarged. The wound was kept open until all hemorrhage had ceased, then brought together by means of sutures, a tent being left in the lower angle of the wound to assist in draining off whatever secretions might collect underneath the flaps. A branch of the temporal artery was cut and ligated. After-treatment consisted simply in changing the dressings twice or three times daily.

In the afternoon of the day on which the operation was made, symptoms of a convulsion came on, but passed off in a moment, leaving no unpleasant sensation behind. It is to be borne in mind that this was the time for the regular monthly attack. He suffered very intensely with headache for the first week, but was kept in a tolerable condition by the use of opiates and iced cloths to the wound. As the pus formed and flowed out through the opening occupied by the tent the pain disappeared, and his progress was very satisfactory, the pain recurring to a greater or less extent each time that the outflow of pus was interfered with. At the end of near four weeks, after being carried some little distance to a relative's house, (this being again about the time for a return of his monthly spasm), he had twitchings of the muscles of the face, but no other signs of a convulsion. For half an hour following the muscular twitchings there was considerable derangement of mind, which, however, left him after this time as clear in mind as before coming on.

Just here comes an interesting feature into the case. A few hours previous to the facial twitchings and slight mental derangement the patient began most violently and continuously to hiccough, lasting seven days, with only an occasional respite of a few hours. So frequent and severe were these hiccoughs that it could be heard at some distance from the house, even with all the windows and doors firmly closed. At times it sounded not unlike the constant barking of a small dog; and whether asleep or awake it was just the same. Dr. Miller saw the case frequently with me, as indeed during his entire sickness, and notwithstanding the use of the numerous and most potent remedies, it continued unchecked, until finally, after seven days, it apparently wore itself out and ceased. Strange to say, aside from the annoyance, the patient did not suffer in the least by this persistent hiccoughing. On the contrary, indeed, for immediately upon its cessation he seemed to be reinvigorated, recovering rapidly both in body and mind. We could discover no connection between this trouble and that about the brain. It seemed to be purely local.

Two days ago, *and for a third time since the operation*, the muscular twitchings of the face, formerly an unfailing evidence of the approach of an epileptic attack, came on and passed away in a moment, with no other effect than leaving the patient in a sleepy condition. This time it came a little in advance of the period at which it was looked for, but this had also been occasionally the case even before the operation. This last, then, is the third regular monthly epileptic attack that my patient has missed since the trephining, now two months since, which had previously not occurred a single time from the moment they began to assume regularity, a year or more ago.

With the exception of keeping the tent in the lower angle of the wound, for the purpose of drainage, nothing is being done for him. In fact, I consider him well, and so also does he consider himself. His general health has been vastly improved, and is still improving. His conversation and his ideas are clearer and more sensible, and that, before mentioned, silly expression of countenance has, to a great extent, disappeared. Only from occasional severe headaches does he now suffer.

No attempt to raise the depressed bone, remaining after the removal of the trephined portion, was made. Now the question naturally arises, was it the removal of the pressure upon the membranes of the brain that caused the attacks of epilepsy to cease? or was it an irritation set up there which, so to speak, drove the disease away? To this latter idea Brown Sequard, in his recent lectures on the nerves, ascribes the good done in such cases. In speaking of epilepsy, he says:

"Another kind of stoppage or arrest of the activity of cells consists in the arrest of the morbid activity of the brain. There are many cases published in the medical journals, relating to insanity, showing that a large number of patients have been cured suddenly by means of irritation of the skin, that was either accidental or employed by a physician. There are other means more curious and equally effective, as in the case I am about to mention. A patient in a lunatic asylum met another one, who struck his head and broke the cranium on the right side. The brain oozed out; a good deal of it was lost, and the patient was cured of his insanity and epilepsy. This is rather a dangerous means of treatment, however, and of course I speak of it only to show that an irritation brought to the brain may often cure. It is in that way that bold surgeons—as many there were in this country in the period from 1825 to 1859—who have brought their instruments to the cranium and made an opening there, in cases of epilepsy, in search of a disease at that place that did not exist, have very frequently cured their patients. But not because they have taken away the disease that they supposed existed there, but because they have produced an irritation which has done it. But I may add that it is not necessary to open the cranium, though it has been done, to my own knowledge, more than fifty times in this country. All that is called for is an irritation of the skin of the cranium and of the fibrous band that covers the bone between the skin and brain. Irritation there has a very good chance of curing epilepsy in many very obstinate cases. There are many cases on record showing that an inflammation in almost any of the organs of the body is sufficient to check insanity. In the same way other affections of the brain, such as amaurosis or paralysis, may be cured suddenly sometimes without any cause that we can find, but with good ground certainly to believe that an irritation has acted which has produced a change in the cells of the brain and diminished their morbid activity. For there are clear cases in which those affections have been cured by such irritation. There is in the brain an irritation starting from the place where there is a disease. That we can see after death. That irritation goes to parts at a distance, and acts on cells to stop their activity; but another irritation, starting from some parts of the body, goes to the parts that are diseased, and there acts on the morbidly active cells and stops their activity, so that the effect that resulted from the disease ceased. So one phenomenon of arrest produced the cessation of another."

Dr. J. M. Johnson, of Atlanta, relates a case, which would appear to suggest the same idea, practiced more than forty years ago.

While the doctor was a student, Dr. Wm. Miller, of Kentucky, his preceptor, who had suffered a number of years with severe sick headaches, ultimately concluded that some pressure upon the membranes of the brain existed at a point in the parietal bone, where a slight natural depression had already existed since childhood. Dr. M. was sure that the depression increased, and in the same proportion did the headaches grow worse. Finally the doctor was stricken with paralysis upon the side opposite to that on which the supposed depression and pressure existed. The most learned physicians of that day were called, even from a great distance, but all their efforts failed to give relief, and each stoutly refused to put into execution the plan again and again urged by the doctor himself, viz: to trephine him, or to apply the *actual cautery* to that portion of the skull supposed to be the seat of trouble. All others refusing, the medical student (the present Dr. J. M. J.) was prevailed upon to make use of the hot iron, which was applied directly to the skin over the natural depression, (for no injury had ever been received), and there held till both patient and student were satisfied that all had been accomplished that could be by the use of such a powerful remedy. After a day or two the patient (Dr. M.) began to improve, continuing until not only the sick headaches and the sense of pressure upon the brain had disappeared, but also until he had thoroughly and rapidly recovered from his paralysis, and was once more as healthy as at any former period of his life. The impression made upon Dr. M.'s mind forty-odd years since, that such an application would cure such a disease, was an idea far in advance of his day, and only of late years has it been revived.

But the great point of inquiry and interest is, is trephining a safe and justifiable remedy to practice for the relief of such diseases? Most assuredly a great risk attends such an operation. Statistics alone well demonstrate this fact. Dudley, who perhaps made the largest number of these operations for the cure of epilepsy, in his report of five cases operated upon previous to 1828, says three were successful. Gross made the next largest number, (four) and reports that three died, and further says that all the other cases upon which he had witnessed operations (three in number), quickly died.

Certainly, as Brown Sequard suggests, if an irritation can be set up, that will answer the same purpose of trephining without any or so many of its attendant dangers, that is the remedy for the disease. In the case reported by myself, it would be reasonably supposed that the attacks would still occasionally occur, becoming gradually less frequent and less severe, until they ultimately ceased altogether. They may recur and run such a course with this case, but judging from the fact that they had previously made their appearance with such persistent regularity, and that since the operation he has passed over three of these periods with nothing more than the merest symptom of a spasm, and also that his health and mind have so much improved since the operation, I am strongly inclined to believe that my patient will become as sound, mentally and bodily, as the most sanguine could hope for.

